# A stratified two-stage tumor molecular profiling algorithm to identify clinically actionable molecular alterations in pancreatic cancer☆

**DOI:** 10.1016/j.esmogo.2025.100134

**Published:** 2025-02-10

**Authors:** S. Hussung, D. Akhoundova, C. Pistoni, D. Lenggenhager, A. Töpfer, C. Pauli, B. Pestalozzi, C. Britschgi, M. Zoche, M. Rechsteiner, H. Moch, A. Weber, R. Fritsch

**Affiliations:** 1 University Hospital Zurich, Department of Medical Oncology and Hematology, Zurich, Switzerland; 2 Faculty of Medicine, University of Zurich, Zurich, Switzerland; 3 Department of Medical Oncology, Inselspital, University Hospital of Bern, Bern, Switzerland; 4 Department for BioMedical Research, University of Bern, Bern, Switzerland; 5 University Hospital Zurich, Department of Pathology and Molecular Pathology, Zurich, Switzerland; 6 Institute of Molecular Cancer Research (IMCR), University of Zurich, Zurich, Switzerland

**Keywords:** pancreatic cancer, tumor molecular profiling, molecular tumor board, personalized treatment, precision oncology

## Abstract

**Background:**

Tumor molecular profiling (TMP) for pancreatic cancer (PC) is recommended by current international guidelines, yet no testing standards exist. Moreover, the magnitude of benefit and the cost-effectiveness of comprehensive next-generation sequencing panels for PC are under debate.

**Materials and methods:**

We implemented a stratified two-stage TMP algorithm for advanced PC. Stage 1 comprised immunohistochemistry for mismatch repair deficiency and targeted sequencing employing a 33-gene next-generation sequencing panel covering common PC drivers and DNA damage response genes. Based on pre-specified events (*KRAS* wild type, mismatch repair deficiency, molecular tumor board recommendation), subsequent comprehensive molecular testing was carried out (stage 2). We report molecular findings and patient outcomes.

**Results:**

A total of 94 PC patients were included in the study. Some 63/94 (67.0%) patients underwent TMP according to the algorithm, of which 5/63 (7.9%) fulfilled criteria for subsequent stage 2 comprehensive testing. A total of 31/94 (33%) patients underwent upfront comprehensive molecular testing outside the algorithm based on referring physician’s request. Compared with algorithm testing, upfront comprehensive testing detected a higher number of pathogenic molecular alterations/patient (median: five versus three, *P* = 0.0005), however no additional actionable alterations. Actionable alterations were identified in 25/94 (26.6%) cases, including DNA damage response gene alterations, KRAS G12C and targetable drivers in *KRAS* wild type tumors. Patients receiving targeted therapy based on molecular profile showed superior survival (progression-free survival, overall survival) compared with patients without targeted treatment.

**Conclusions:**

Stratified two-stage TMP reliably identifies actionable alterations in PC patients, with potential therapeutic benefit. The proposed TMP algorithm might be as effective, yet more feasible and economic compared with comprehensive upfront testing.

## Highlights

• We established a stratified two-stage tumor molecular profiling (TMP) algorithm for advanced pancreatic cancer (PC).
• Algorithm testing identified a similar number of clinically actionable alterations compared with comprehensive testing.
• Patients receiving targeted therapy based on TMP showed improved survival outcomes.
• Stratified two-stage TMP is a highly feasible and effective alternative to upfront comprehensive molecular profiling in PC.

## Introduction

Pancreatic cancer (PC) is a common tumor entity characterized by a dismal prognosis and a high impact on global cancer mortality.^1^ PC is characterized by an aggressive tumor biology, an immunosuppressive tumor microenvironment, and a challenging molecular build-up with a high prevalence of undruggable genomic alterations.^2^, ^3^, ^4^, ^5^, ^6^ In the clinic, systemic chemotherapy remains the mainstay of PC treatment, with little progress in targeted treatment achieved over the recent decade. At the foremost, PC poses a major challenge for precision oncology, given that robust predictive biomarkers for treatment selection and clinically actionable molecular alterations can only be identified in up to 25% of the cases.^7^, ^8^, ^9^, ^10^, ^11^

Clinically actionable genomic alterations in metastatic PC include (i) alterations in DNA damage response (DDR) genes,^9^^,^^12^ (ii) KRAS G12C driver mutations,^13^, ^14^, ^15^ (iii) microsatellite instability-high (MSI-H), and (4) *KRAS* wild type (*KRAS*^*WT*^) tumors harboring rare targetable driver mutations, including class 1 and 2 *BRAF* alterations,^16^ as well as oncogenic fusions.^17^

Genomic alterations in DDR genes are found in ∼20% of PCs, mostly within the homologous recombination repair (HRR) pathway. Enhanced sensitivity to platinum-based chemotherapy has been demonstrated for PCs with deleterious HRR gene alterations,^3^^,^^18^, ^19^, ^20^, ^21^, ^22^, ^23^, ^24^, ^25^ constituting the currently best-established predictive biomarker for first-line systemic chemotherapy selection in the clinic.^26^ Moreover, vulnerability to poly(ADP-ribose)polymerase (PARP) inhibitors is a hallmark of PCs with pathogenic *BRCA1/2* alterations.^27^ Based on the results of the phase III POLO trial, olaparib was approved as maintenance therapy for patients with metastatic PC and germline *BRCA1/2* alterations.^9^ The biological and clinical significance of germline or somatic core HRR gene alterations, typically defined as loss-of-function mutations and genomic deletions of *BRCA1*, *BRCA2* or *PALB2*, are widely accepted.^26^^,^^28^ The role of non*-BRCA* DDR alterations,^19^ however, remains less well defined,^29^, ^30^, ^31^, ^32^ with recent data pointing to enhanced platinum sensitivity of PCs harboring non-core DDR gene alterations.^20^^,^^31^^,^^33^, ^34^, ^35^, ^36^, ^37^

KRAS G12C inhibitors have shown promising efficacy in PC, however, they might induce not as durable responses as observed for other tumor entities, including non-small-cell lung cancer.^13^^,^^15^^,^^38^, ^39^, ^40^ A very small subset of PCs (<1%) show mismatch repair deficiency (dMMR).^41^^,^^42^ These tumors are enriched in patients with germline MMR gene mutations and show sensitivity to treatment with anti-programmed cell death protein 1 immune checkpoint inhibitors.^43^^,^^44^ *KRAS*^WT^ PCs harbor a set of rare, highly targetable oncogenic driver alterations, including class 1 and 2 *BRAF* alterations^16^ and fusion oncogenes including *NRG1*,^17^ *NTRK*,^45^ *FGFR2*,^46^ *BRAF, RET*, and others.^47^

Based on these recent advances in PC precision treatment, tumor molecular profiling (TMP) is now recommended for all patients with locally advanced or metastatic PC for systemic treatment by National Comprehensive Cancer Network (NCCN) and ESMO guidelines,^26^^,^^48^ in addition to *BRCA* germline testing.^28^ No testing standards, however, have been established to date.^28^ Moreover, clinical implementation and reimbursement of PC TMP varies widely between individual countries.^49^

We established a two-stage PC TMP algorithm at our institution, employing a targeted dedicated 33-gene next-generation sequencing (NGS) panel combined with MMR immunohistochemistry (IHC) for all PC patients, followed by subsequent comprehensive testing in a molecularly selected patient population. The algorithm aims to reliably identify clinically actionable molecular alterations, while avoiding the higher costs and tissue requirements of carrying out upfront comprehensive molecular testing without evidence for significant additional benefit.

## Material and methods

### Patient cohort

Patients with clinically and histologically or cytologically confirmed adenocarcinoma of the pancreas who underwent TMP at the University Hospital Zurich (USZ) between January 2019 and June 2022 were included in the study. Neuroendocrine neoplasias, as well as rare tumor subtypes, including acinar cell carcinomas and mixed histologies, were excluded from the analysis. Diagnosis of locally advanced or metastatic disease was based on radiological and cytological/histological confirmation. All patients provided written informed consent to the collection and analysis of genomic and clinical data, which were extracted from electronic medical records. The local institutional review board approved all relevant procedures and analyses (Cantonal Ethics Committee Zurich: BASEC No. 2021-01094). The study was conducted in accordance with the Good Clinical Practice guidelines and the Declaration of Helsinki. Systemic treatment was carried out as per standard of care. Molecular profiling results were discussed at our institution’s weekly molecular tumor board (MTB). Routine follow-up included 2- to 3-monthly clinical and radiological examination using computed tomography and/or magnetic resonance imaging. Radiological response evaluation was based on routine evaluation of radiological imaging.

### Derivation of a 33-gene PC NGS panel

**Figure 1:**
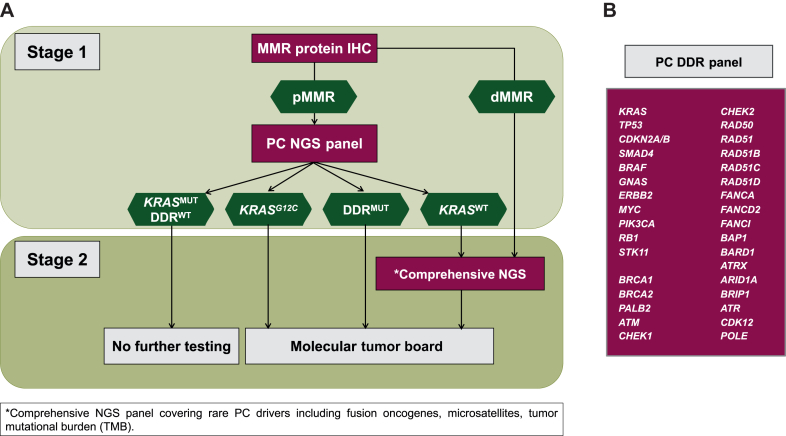
**PC two-stage molecular profiling algorithm.** (A) Schematic illustration of the algorithm workflow. Stage 1 consists of PC-customized 33-gene NGS panel and IHC for MMR proteins. Patients with *KRAS*^WT^ or dMMR, as well as selected patients upon molecular tumor board recommendation, undergo stage 2 comprehensive NGS. (B) 33-Gene NGS panel, including most frequently altered PC-driver and DDR genes. DDR, DNA damage response; dMMR, mismatch repair deficient; IHC, immunohistochemistry; MMR, mismatch repair; MUT, mutant; NGS, next-generation sequencing; PC, pancreatic cancer; pMMR, mismatch repair proficient; WT, wild type.

A customized 33-gene NGS panel (‘PC DDR panel’) was derived from the Oncomine Comprehensive Assay v3 (Thermo Fisher Scientific, Waltham, MA). This NGS panel detects single nucleotide variants (SNV) and copy number variations (CNV) for 22 DDR genes plus 11 disease-relevant cancer genes including the most commonly altered oncogenes and tumor suppressors of PC (Figure 1B). The following 22 DDR genes were included in the PC DDR panel: *BRCA1*, *BRCA2*, *PALB2*, *ATM*, *CHEK1*, *CHEK2*, *RAD50*, *RAD51*, *RAD51B*, *RAD51C*, *RAD51D*, *BAP1*, *BARD1*, *ATRX*, *ARID1A*, *BRIP1*, *ATR*, *FANCA*, *FANCD2*, *FANCI*, *CDK12*, and *POLE*. For data analysis, based on current literature, we subdivided DDR genes in core-alterations (*BRCA1*, *BRCA2*, and *PALB2*) and non-core (*ATM*, *CHEK1*, *CHEK2*, *RAD50*, *RAD51*, *RAD51B*, *RAD51C*, *RAD51D*, *BAP1*, *BARD1*, *ATRX*, *ARID1A*, *BRIP1*, *ATR*, *FANCA*, *FANCD2*, *FANCI*, *CDK12*) alterations.

### NGS

Tumor DNA was isolated using Maxwell® 16 DNA Purification Kits (Promega, Madison, WI). DNA concentration was quantified using a Qubit® Fluorometer (Thermo Fisher Scientific, Waltham, MA). For the PC DDR NGS panel, emulsion PCR, enrichment and loading of the chip were carried out on the Ion Chef™ System (Thermo Fisher Scientific) with the Ion 520/530™ Kit or 540™ Kit – Chef. Sequencing was carried out on the Ion S5™ platform with the Ion S5™ Sequencing Kit. All steps were carried out according to manufacturer’s instructions. Alignment, variant calling, and annotation were carried out with the Ion Reporter™ Software 5.14. FoundationOne®CDx is an FDA-approved targeted NGS assay identifying base pair substitutions, insertions and deletions, as well as copy number alterations in 324 genes and genomic rearrangements in 36 selected genes.^50^ In addition, MSI status, and tumor mutational burden (TMB) were reported. Hybrid capture-selected libraries, generated from 50-1000 ng DNA isolated from formalin-fixed paraffin-embedded (FFPE) tumor tissue specimens, were sequenced using an IlluminaR HiSeq platform. Sequencing data were processed using a customized analysis pipeline. All NGS diagnostics are accredited at the Department of Pathology and Molecular Pathology at USZ.

### Tissue processing and MMR IHC

FFPE tumor tissue sections (2 μm) were used to carry out IHC of MMR proteins (MLH1, MSH2, MSH6, and PMS2). The following antibodies were used: anti-MLH1 (clone ES05, Novocasta Laboratories Ltd., Newcastle upon Tyne, UK, dilution 1 : 40), anti-PMS2 (clone EP51, Leica Biosystems, Nussloch, Germany, ready to use mix), anti-MSH6 (clone SP93, Cell Marque Lifescreen Ltd., Rocklin, CA, ready to use mix), anti-MSH2 (clone G219-1129, Cell Marque Lifescreen Ltd, dilution 1 : 100). Samples were classified as mismatch repair proficient (pMMR) if nuclear staining was detected in >10% of tumor cells.

### Study endpoints and statistical analyses

Primary endpoint was the frequency of clinically actionable molecular alterations detected by TMP.^51^ Clinical actionability was defined as targetability in label or off-label with Food and Drug Administration (FDA)-approved drugs, associated with expected clinical benefit. Secondary endpoints included progression-free survival (PFS), overall survival (OS), and systemic treatment choice. PFS was defined as time from start of systemic treatment of locally advanced or metastatic disease to the first radiologic progression or death due to any cause. OS was defined as time from first diagnosis of locally advanced or metastatic disease to death. Patients were censored on the last follow-up date or latest on 8 June 2022. Targeted treatment was defined as the (i) selection of a platinum-containing chemotherapy regimen based on molecular profile, (ii) treatment with PARP inhibitors, (iii) KRAS G12C inhibitors or (iv) other targeted treatment based on molecular profile. Descriptive statistical analyses were carried out for categorical and continuous variables of interest. Kaplan–Meier survival analyses were carried out to estimate PFS and OS probabilities. Univariate analyses were carried out using the log-rank test. Backward stepwise Cox regression modeling to estimate hazard ratio (HR) with 95% confidence interval (CI) was used to explore independent prognostic factors for PFS and OS. All statistical analyses were carried out using GraphPad Prism Version 9.5.1 (GraphPad Software, Inc., La Jolla, CA) and SPSS 26 software Version 26.0.0.0 (IBM Corporation, Armonk, NY). All *P* values were two-sided with *P* < 0.05 considered as statistically significant.

## Results

### Patient cohort

A total of 94 patients with histologically confirmed PC adenocarcinoma, treated at USZ between January 2019 and June 2022 were included in the study. Clinical and pathological baseline characteristics are summarized in Supplementary Table S1, available at https://doi.org/10.1016/j.esmogo.2025.100134. Most frequent histology was pancreatic ductal adenocarcinoma (95.7%). Some 88/94 (93.6%) patients had unresectable or metastatic disease. A total of 6/94 (6.4%) patients underwent TMP for resectable disease and were included for primary endpoint analysis only, but not for treatment and survival analyses. A total of 76/88 (86.4%) patients with unresectable and metastatic disease received first-line chemotherapy, 10/88 (11.4%) underwent best supportive care, 2/88 (2.3%) were lost to follow-up. Some 33/88 (37.5%) patients received platinum-based first-1st line chemotherapy with FOLFIRINOX (*n* = 31) or FOLFOX (*n* = 2) (Supplementary Table S1, available at https://doi.org/10.1016/j.esmogo.2025.100134). Median follow-up for patients was 13.5 months (range: 0.3-39.0 months). Median first-line PFS was 6.0 months (95% CI 4.0-7.0 months), median OS was 15.0 months (95% CI 10.0-17.0 months) (Supplementary Figure S1A and B, available at https://doi.org/10.1016/j.esmogo.2025.100134). The proportion of patients receiving second- and third-line systemic treatment is illustrated in Supplementary Figure 1C, available at https://doi.org/10.1016/j.esmogo.2025.100134. At the time of final analysis, 63/88 (71.6%) of the patients had died.

### Stratified two-stage molecular profiling algorithm

We established a two-stage TMP algorithm specifically for PC. In stage 1, all tumor samples underwent IHC for MMR proteins (MLH1, MSH2, MSH6, and PMS2). In all patients with pMMR tumors, a customized 33-gene NGS panel was carried out (Figure 1A). The 33-gene NGS panel detects SNV and CNV for 22 DDR genes plus 11 disease-relevant cancer genes including the most commonly altered oncogenes and tumor suppressors of PC (Figure 1B).

According to the algorithm, additional comprehensive molecular testing with the Foundation One® CDx targeted NGS assay (stage 2) was carried out for tumors with unmutated *KRAS* (*KRAS*^*WT*^), dMMR, or upon request of the MTB. All medical oncologists treating PC patients within the department were trained on the algorithm. Due to institutional policy and the reimbursement situation in Switzerland, however, oncologists remained free to order either algorithm testing or upfront comprehensive testing at their discretion.

### Adherence to TMP algorithm

**Figure 2:**
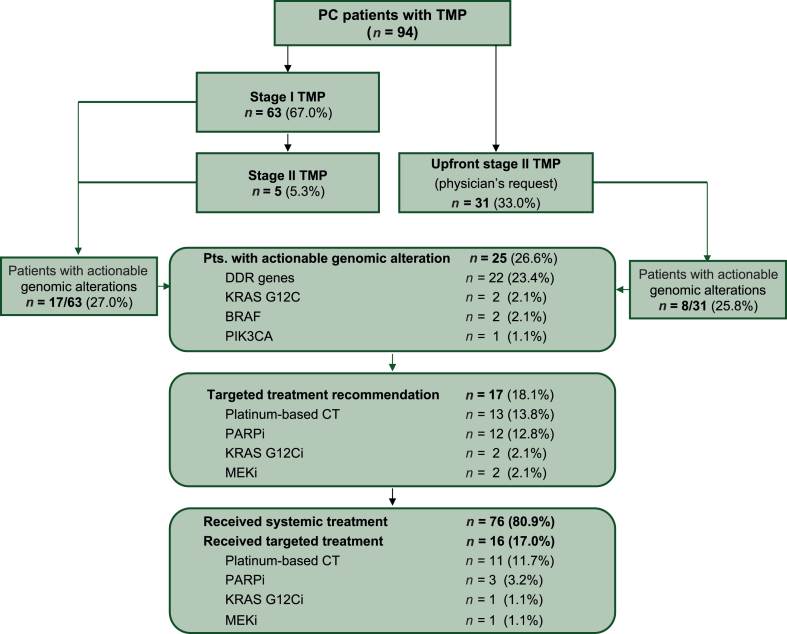
Diagram illustrating PC adenocarcinoma patient workflow within the TMP algorithm. BSC, best supportive care; CT, chemotherapy; DDR, DNA damage response; i, inhibitors; PARP, poly (ADP-ribose) polymerase; PC, pancreatic cancer; Pts., patients; syst., systemic; TMP, tumor molecular profiling.

A total of 63/94 (67.0%) patients underwent molecular testing according to the TMP algorithm, of whom 5/63 underwent stage 2 comprehensive testing (FoundationOne®CDx) after stage 1 based on pre-specified criteria (Figure 2). The remaining 31/94 (33.0%) patients underwent upfront comprehensive testing (FoundationOne®CDx) outside the TMP algorithm based on physician’s request (Figure 2). Molecular testing was carried out on archived resection specimens (34/94, 36.2%), fine needle aspirates (33/94, 35.1%), and core needle biopsies (23/94, 24.5%) (Supplementary Table S1, available at https://doi.org/10.1016/j.esmogo.2025.100134). The median estimated tumor cell content across all samples was 35% (95% CI 30.0% to 40.0%). In 42/94 cases (44.7%), molecular testing was initiated before first-line systemic treatment.

### Molecular landscape and frequency of clinically actionable alterations

**Figure 3:**
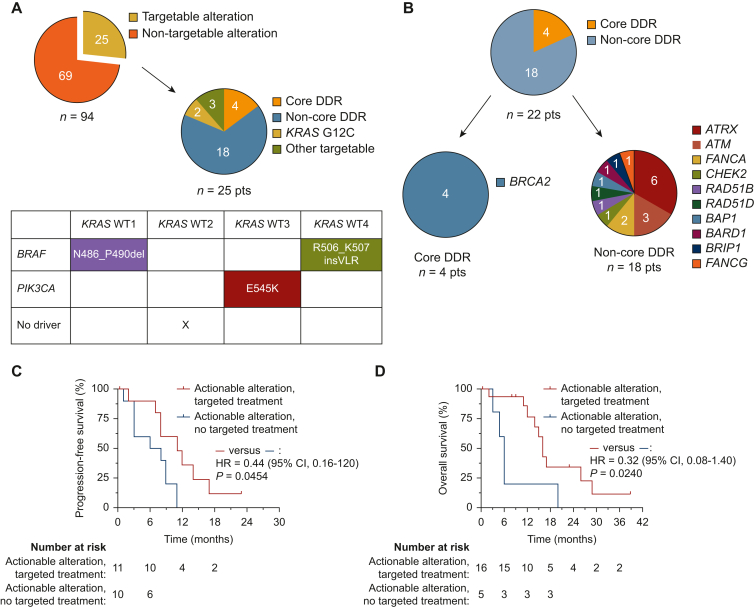
**Actionable alterations and association with survival outcome**. (A) The frequencies and distribution of actionable alterations within the PC cohort are illustrated. Table below shows actionable genomic alterations detected by TMP within the four *KRAS* wild type cases. (B) Frequency and distribution of core and non-core DDR alterations within the cohort. (C, D) Survival of patients with unresectable and metastatic PC in the cohort. PFS (C) and OS (D) are shown for two patient subgroups: patients with actionable alterations who received targeted treatment and patients with actionable alterations who did not receive targeted treatment. CI, confidence interval; DDR, DNA damage response; HR, hazard ratio; OS, overall survival; PC, pancreatic cancer; PFS, progression-free survival.

In 94 patients, we identified a total of 347 molecular alterations reported as pathogenic or likely pathogenic in molecular pathology reports, with a median of 3 alterations per patient for algorithm patients and 5 alterations in patients with upfront comprehensive testing (*P* = 0.0005) (Figure 2). As expected, most common molecular alterations were detected in *KRAS* (90/94; 95.7%), of which 34/90 (38.7%), 34/90 (38.7%), 12/90 (13.3%), and 2/90 (2.2%) were KRAS G12V, KRAS G12D, KRAS G12R, and KRAS G12C, respectively. Other common molecular alterations were detected in *TP53* (63/94; 67.0%), *CDKN2A/B* (39/94; 41.5%) and *SMAD4* (19/94; 20.2%) (Supplementary Figure S2, available at https://doi.org/10.1016/j.esmogo.2025.100134 for full molecular findings). All tumors were pMMR (94/94, 100%). In total, we identified 25/94 patients (26.6%) with at least one clinically actionable molecular alteration, most frequently within the DDR gene set (22/94) (Figure 3A and B). Other clinically actionable alterations were KRAS G12C (2/94; 2.1%), class II BRAF alterations in 2/4 *KRAS*^WT^ patients (Figure 3A and B), and a *PIK3CA* hotspot mutation in 1/4 *KRAS*^WT^ patients. In 0/31 cases, upfront comprehensive testing uncovered targetable alterations outside the coverage of the two-stage algorithm (Figure 2 and Supplementary Figure S2, available at https://doi.org/10.1016/j.esmogo.2025.100134).

### Personalized treatment recommendations by MTB

Some 80/94 (85.1%) cases were discussed at our institution’s MTB. A personalized treatment recommendation based on TMP was given in 17/94 (18.1%) patients. A total of 11/17 recommendations were clinically implemented, i.e. the recommended treatment given to patients (Figure 2). Specifically, platinum-based chemotherapy was recommended in 13/94 patients (13.8%), and implemented in 8/94 patients (8.5%) based on alterations within the DDR gene subset (Figures 2 and 3A and B). PARP inhibitors (in-label or off-label) were recommended in 12/94 (12.8%) cases, but implemented only in 3/94 cases (3.2%). Off-label targeted treatment of KRAS G12C (sotorasib) was recommended in 2/94 (2.1%) cases and implemented in 1 case. Similarly, targeted treatment with a mitogen activated protein kinase inhibitor for class II *BRAF* alterations found in *KRAS*^WT^ tumors were recommended in 2/94 (2.1%) cases and given to one patient (Figure 2). Additional germline testing beyond *BRCA1/2* was recommended in 6/94 (6.4%) patients based on molecular profiling results.

### Algorithm testing versus upfront comprehensive NGS

When cross-comparing patients undergoing algorithm testing with patients undergoing upfront comprehensive testing with FoundationOne®CDx, a significantly higher number of pathogenic molecular alterations were detected with comprehensive testing (median of 5 versus 3 pathogenic alterations per patients, *P* = 0.0005). Importantly, however, not a single personalized treatment recommendations in the comprehensive testing group was based on alterations outside the coverage of the TMP algorithm (0/31 cases). While upfront comprehensive testing did uncover additional potentially targetable alterations in some cases including *FGFR3* and *AKT2* amplifications (Supplementary Figure S2, available at https://doi.org/10.1016/j.esmogo.2025.100134), these alterations occurred in *KRAS*-mutated tumors and were therefore judged not clinically actionable by MTB (not shown).

### Targeted treatment and survival endpoints

Of 22 patients with actionable alterations, 11 patients received targeted treatment first line, while 10 did not (Figure 3C). One patient presented with resectable disease and was therefore not included in survival analyses. Patients receiving targeted treatment first line achieved superior median PFS when compared with patients with actionable alterations and no targeted first-line treatment [11 months versus 7 months; HR 0.44 (95% CI 0.16-1.20; *P* = 0.0454); Figure 3C]. Also, despite very small numbers, patients receiving targeted treatment based on TMP in any treatment line showed superior median OS compared with patients with actionable alterations who did not receive targeted treatment [16 months versus 6 months; HR 0.32 (95% CI 0.08-1.40; *P* = 0.024)]. When excluding patients with DDR alterations receiving platinum, a total of five patients received specific targeted treatment. While the number of patients was exceedingly small, the exploratory OS analysis suggested a potential advantage for those receiving targeted treatment compared with patients with actionable alterations who did not receive targeted treatment [median OS 32.5 versus 13 months; HR 0.11 (95% CI 0.01-2.09), *P* = 0.0193; Supplementary Figure S1D, available at https://doi.org/10.1016/j.esmogo.2025.100134].

### Personalized treatment and disease course

**Figure 4:**
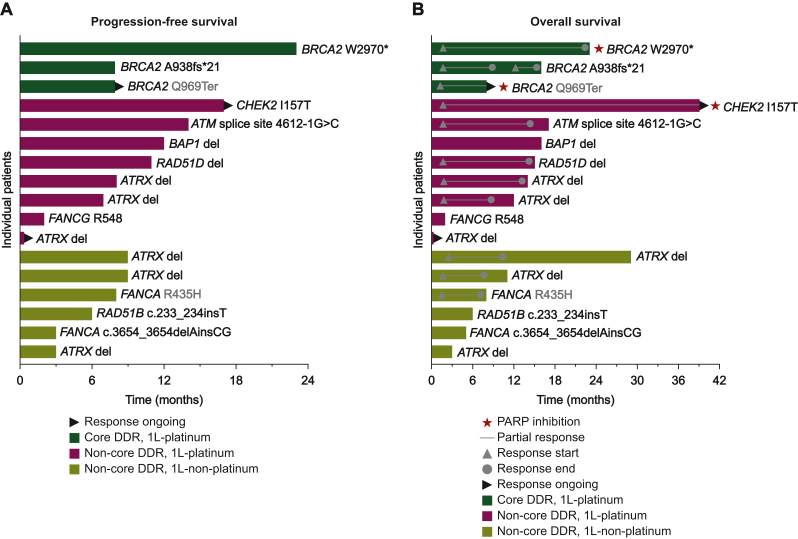
**DDR alterations and association with survival outcome**. Swimmer plots of first-line PFS (A) and OS (B) of PC cancer patients with core and non-core DDR alterations. 1L, first-line; DDR, DNA damage response; OS, overall survival; PARP, poly (ADP-ribose) polymerase; PC, pancreatic cancer; PFS, progression-free survival.

**Figure 5:**
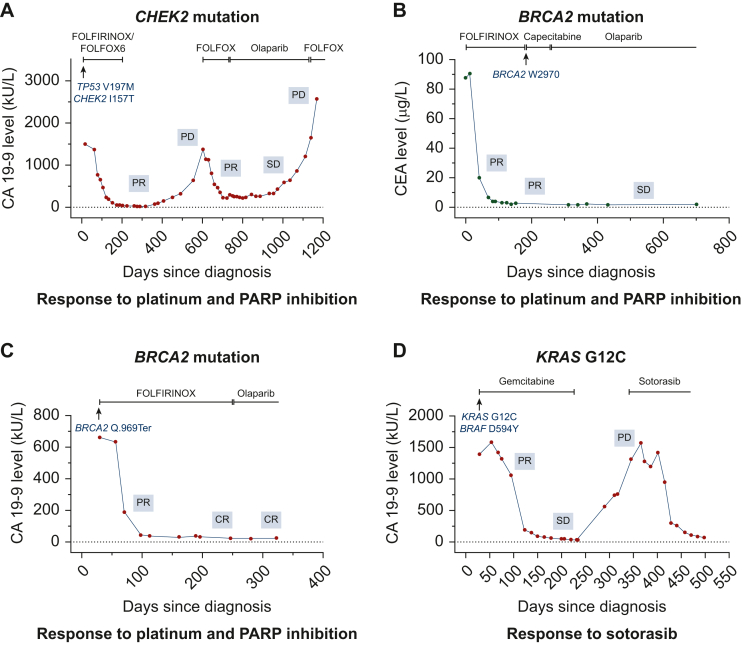
**Patient case reports.** The tumor marker course, together with radiological responses and applied systemic treatments are depicted for four individual cases of metastatic PC with actionable alterations detected by TMP. (A) A 41-year-old female PC patient with suspected Li-Fraumeni syndrome with concomitant *TP53* (VAF 77%) and *CHEK2* (VAF 50%) mutation. The patient received three courses of platinum-based chemotherapy and one course of olaparib maintenance therapy. (B) A 65-year-old male PC patient with a *BRCA2* mutation (VAF 51%, germline diagnostics declined) detected by molecular tumor testing, with prolonged partial response (PR) to platinum-based chemotherapy and maintenance with olaparib. (C) A 65-year-old male PC patient with germline *BRCA2* mutation (VAF 56%), showing sustained response to platinum-based chemotherapy, followed by olaparib maintenance. (D) A 79-year-old female patient with KRAS G12C mutations (VAF 9.7%), achieved a 3-month PR to targeted treatment with sotorasib. CEA, carcinoembryonic antigen; PARP, poly(ADP-ribose)polymerase; PC, pancreatic cancer; PD, progressive disease; SD, stable disease; TMP, tumor molecular profiling; VAF, variant allele fraction.

The disease courses of individual patients with DDR alterations exposed to platinum-based or platinum-free therapy, including first-line PFS and OS, are illustrated in Figure 4A and B. Despite heterogeneous responses, patients with DDR-mutated PC overall appeared to derive a benefit from platinum-based first-line treatment (Figure 4A), with some exceptional responders and long-term survivors (Figure 4B). Figure 5 illustrates the detailed disease courses of index patients receiving targeted treatment based on TMP. Figure 5A shows an exceptional response to platinum-based first-line treatment, platinum re-exposure, and subsequent PARP inhibition with olaparib in a patient with a *CHEK2* mutation. Figure 5B and C illustrates responses to platinum and olaparib in two patients with pathogenic *BRCA2* mutations. Figure 5D shows off-label treatment of a KRAS G12C-mutated PC with sotorasib. Another patient of this cohort harboring a class II *BRAF* mutation in a *KRAS*^WT^ PC has been reported previously, including the response to targeted treatment with trametinib.^52^ Lastly, we analyzed whether there were independent demographic and clinic-pathologic features predictive for PFS and OS. Univariate analysis showed that the presence of DDR alterations was associated with improved PFS: HR = 0.60; 95% CI 0.36-0.98 (*P* = 0.05) (Supplementary Table S2, available at https://doi.org/10.1016/j.esmogo.2025.100134). Moreover, the multivariate Cox proportional hazards regression model showed that targeted treatment was the only variable associated with longer OS: HR = 0.07; 95% CI 0.011-0.435 (*P* = 0.004) (Supplementary Table S2, available at https://doi.org/10.1016/j.esmogo.2025.100134).

## Discussion

The emerging therapeutic success of molecularly targeted treatment in PC supports early molecular profiling in all PC patients undergoing active treatment.^9^^,^^13^^,^^28^^,^^53^ The optimal molecular profiling strategy, however, has yet to be defined. In clinical practice, TMP in PC patients can be technically challenging due to sparse material (often fine-needle aspirations) and low tumor cell content. Also, based on treatment limitations including the lack of targeted agents for the most prevalent KRAS SNVs, only a subset of patients currently derives therapeutic benefit from PC TMP. Both limitations, together with the soaring costs of cancer care, in our view, argue against carrying out large comprehensive NGS panels upfront in all PC patients. In our study, we proposed a stratified two-stage testing approach, aiming for a highly feasible and cost-effective alternative to upfront comprehensive testing. Our data show that stratified molecular testing is feasible in the clinical routine and uncovers potentially targetable molecular alterations in a relevant subset of patients. Moreover, while testing according to the proposed stratified algorithm did identify fewer pathogenic molecular alterations, it did not miss actionable alterations compared with upfront comprehensive testing. Additionally, compared with more comprehensive NGS panels, our customized DDR panel is associated with lower assay costs, being suitable as well for serial analysis repeated through disease course. Similar results with employing a small upfront NGS panel, followed by selective more comprehensive testing in selected cases, were reported recently from a retrospective German cohort.^54^ Our data confirm these findings and, by prospectively establishing a two-stage TMP algorithm, we provide proof of clinical feasibility of such an approach. An important limitation of our study, however, is that ∼33% of patients underwent upfront comprehensive molecular testing outside of the proposed algorithm. This deviation primarily resulted from the choices made by referring physicians, who retained final decision-making authority regarding molecular testing. Despite all treating oncologists receiving training on the new algorithm, some were initially hesitant to adopt it, particularly during the early phase of implementation. Therefore, we cannot entirely exclude the possibility that clinical factors influenced the decision to carry out upfront comprehensive testing in certain cases, representing a potential source of bias. Even within our small cohort, we observed longer first-line PFS and OS for PC patients receiving targeted treatment based on molecular profiles. This is in agreement with previously reported cohorts^19^ and underlines the importance of carrying out upfront TMP in PC patients. Targeted first-line treatment in PC currently means platinum-based chemotherapy. Guidelines support the use of the FOLFIRINOX protocol or cisplatin–gemcitabine (NCCN guidelines^48^) in patients with core homologous recombination deficiency (HRD) alterations, whenever clinically feasible. In a field characterized by the lack of predictive biomarkers, limited therapeutic options, and little progress,^55^ genotype-guided choice of first-line therapy appears crucial.^7^^,^^56^ It is important to note that while our observations align with previously published data, interpreting the survival analyses in our study remains challenging due to the low number of patients in the different subgroups. Therefore, our findings regarding longer PFS and OS data should be considered exploratory and hypothesis generating only. In addition, several questions remain unsolved. For instance, to date no consensus exists as to which DDR genes should be considered as ‘targetable’ and how to optimally assess HRD in PC.^37^^,^^57^ We omitted any direct assessment of genomic instability though quantitative measurement of HRD scores, which typically include loss of heterozygosity, large scale transitions, and telomeric allelic imbalances scores. Although technically feasible, there currently is, in our view, insufficient evidence to draw therapeutic conclusions from these scores for PC treatment.

Our MTB recommended off-label PARP inhibition in all patients with core and several patients with non-core DDR alterations. In PC, there is a lack of prospective evidence supporting PARP inhibition in DDR-altered tumors beyond *BRCA1* and *BRCA2* alterations.^9^ In our view, however, off-label PARP inhibition can be discussed on an individual basis in patients with non-core HRR gene alterations and excellent response to platinum-based chemotherapy as shown in Figure 5A.

A further question under debate is the optimal relationship between somatic and germline testing in PC patients. While upfront germline testing is recommended in all PC patients by NCCN and several national guidelines, this approach has not been uniformly adopted across Europe and is not feasible in all countries.^48^ Moreover, from a therapeutic standpoint, TMP will identify both targetable somatic and germline alterations and in many countries has faster turnover times compared with germline testing. Still, it is crucial to point out that TMP can never replace germline testing, and that both tests are indicated in PC patients.

Small study cohort, lack of randomization, and retrospective analyses of clinical outcomes constitute limitations of our study. Results presented in this analysis are therefore of an exploratory nature and hypothesis generating. In summary, we advocate stratified tumor tissue testing for all stage IV PC patients, ideally, before start of first-line treatment. Treatment selection based on molecular monitoring appears feasible, but still requires extensive clinical validation in interventional studies.

### Conclusions

Routine stratified molecular testing for PC is feasible and identifies targetable molecular alterations in a relevant subset of cases, most commonly alterations within the DDR gene subset. Patients receiving targeted treatment based on molecular profiling show an improved outcome. Based on these results, we recommend up-front molecular tumor testing for all stage IV PC patients; however, the best possible test strategy remains to be established. A stratified two-stage testing approach might be a more feasible and efficient alternative to upfront comprehensive testing.
