# Supplementary figures and images for: A stratified two-stage tumor molecular profiling algorithm to identify clinically actionable molecular alterations in pancreatic cancer
Source: ESMO Gastrointest Oncol. 2025 Feb 10;7:100134. doi: 10.1016/j.esmogo.2025.100134 (PMC12836705; doi:10.1016/j.esmogo.2025.100134)

A

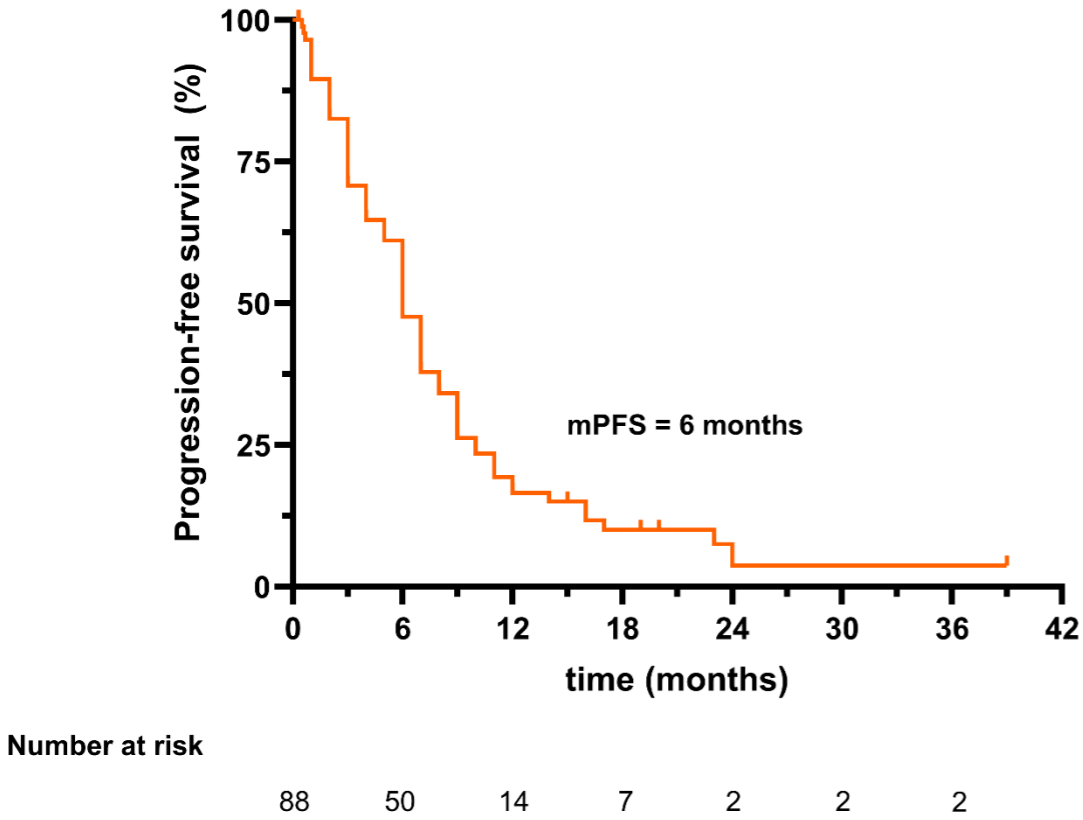

B

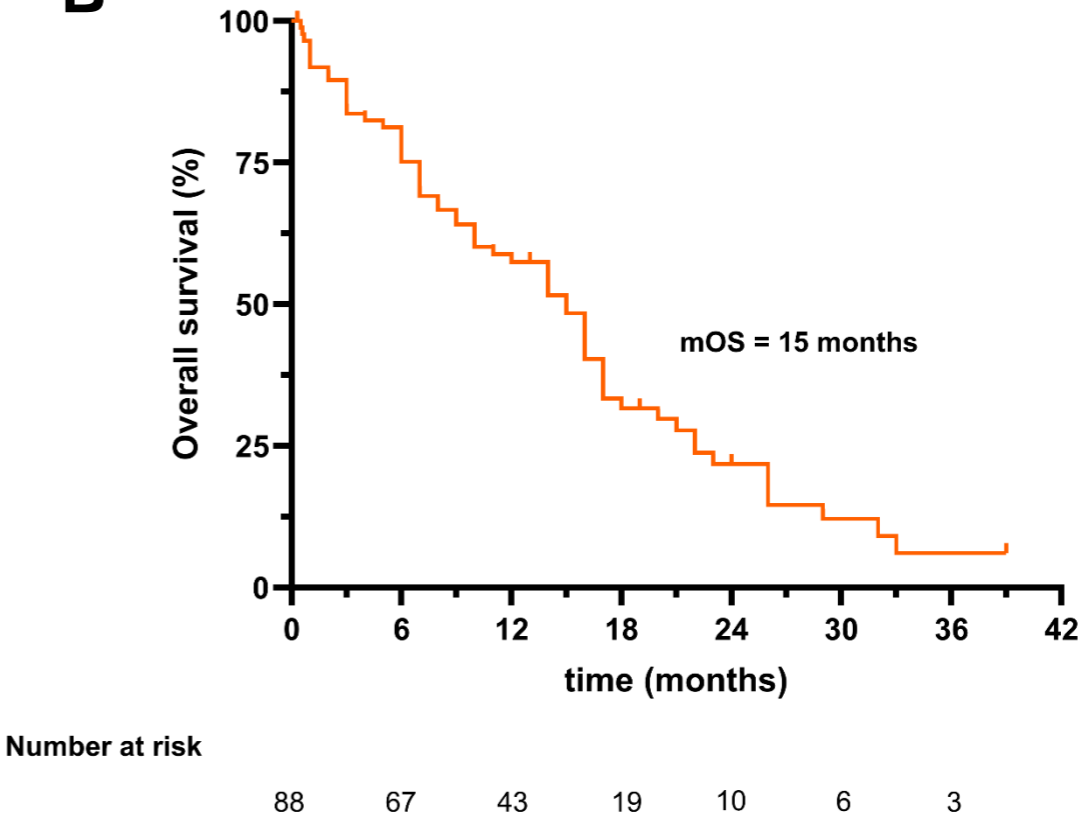

C

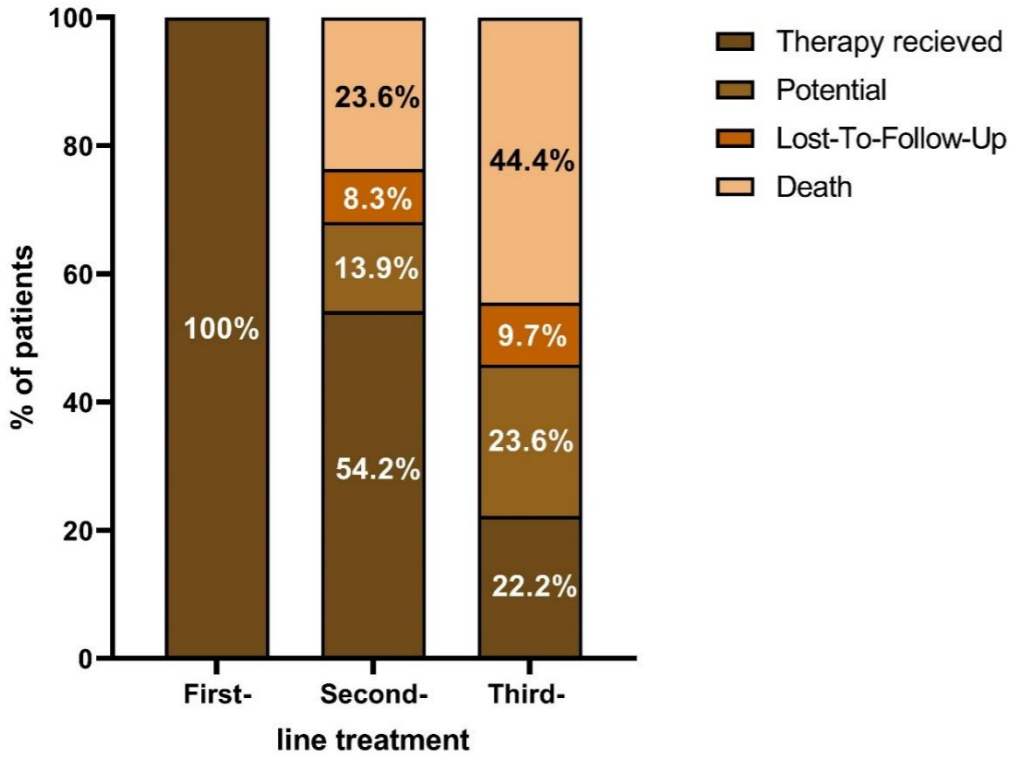

D

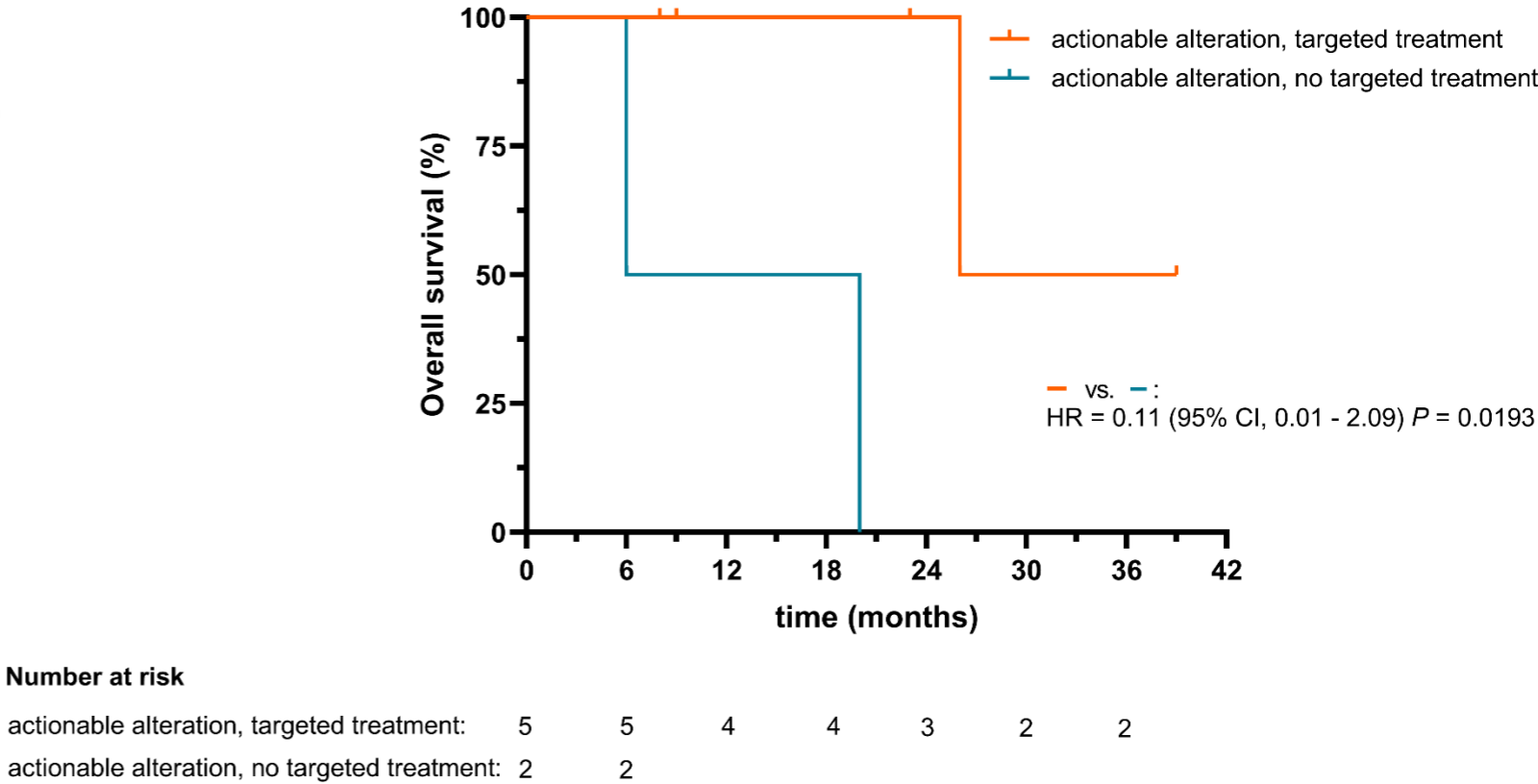

Supplement: Supplemental Figure 1 [file mmc1.pdf]

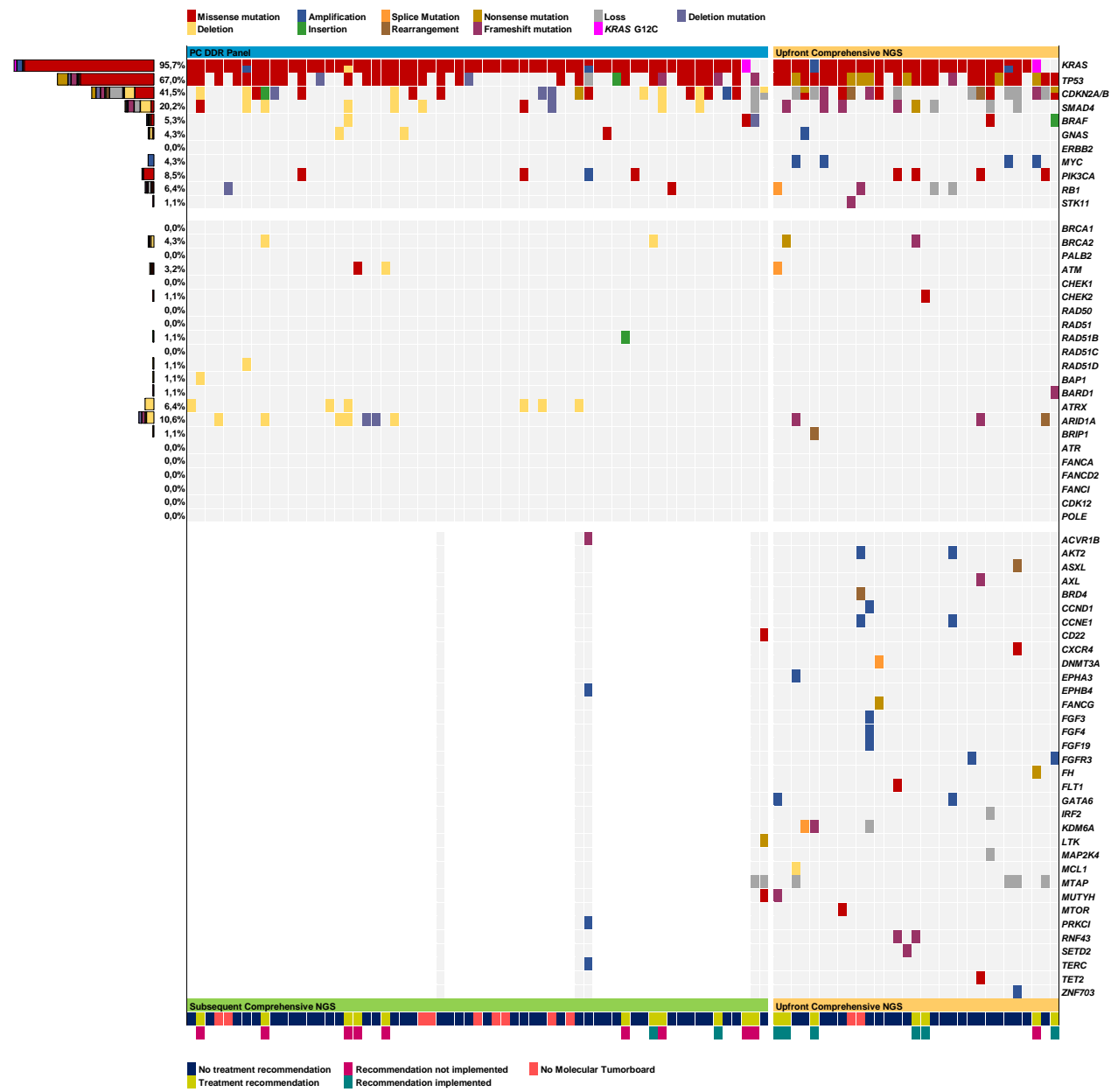

Supplement: Supplemental Figure 2 [file mmc2.pdf]
