# Supplementary material for: A stratified two-stage tumor molecular profiling algorithm to identify clinically actionable molecular alterations in pancreatic cancer
Source: ESMO Gastrointest Oncol. 2025 Feb 10;7:100134. doi: 10.1016/j.esmogo.2025.100134 (PMC12836705; doi:10.1016/j.esmogo.2025.100134)
Supplement: Supplemental Table 1 [file mmc3.docx]

**Supplemental Table 1. Clinico-pathological parameters and basic clinical characteristics of the study cohort (n = 94).**

|  | Number of patients | Percentage (%) |
| --- | --- | --- |
| Gender  Male  Female | 64  30 | 68.1  31.9 |
| Pathology  Ductal adenocarcinoma  Carcinoma, NOS | 90  4 | 95.7  4.3 |
| Location of primary  Head  Body  Tail  Overlap | 50  14  16  14 | 53.2  14.9  17.0  14.9 |
| Stage at molecular testing  Resectable  Locally advanced  Metastatic | 6  18  70 | 6.4  19.1  74.5 |
| Material for molecular testing  Resection specimen  Fine needle aspiration  Core needle biopsy  Other | 34  33  23  4 | 36.2  35.1  24.5  4.3 |
| First-line Treatment (n=88)  FOLFIRINOX  Gemcitabine/nab-Paclitaxel  FOLFOX  Gemcitabine mono  Other  BSC  Incomplete data  Lost to follow-up | 31  30  2  4  5  10  4  2 | 35.2  34.1  2.3  4.5  5.7  11.4  6.8  2.3 |
|  | **Median** | **Range (95% CI)** |
| Age (years) | 67 | 30 - 86 (64 - 70) |

CI, Confidence interval
