# Supplementary material for: A stratified two-stage tumor molecular profiling algorithm to identify clinically actionable molecular alterations in pancreatic cancer
Source: ESMO Gastrointest Oncol. 2025 Feb 10;7:100134. doi: 10.1016/j.esmogo.2025.100134 (PMC12836705; doi:10.1016/j.esmogo.2025.100134)
Supplement: Supplemental Table 2 [file mmc4.docx]

**Supplemental Table 2. Uni- and multivariate analyses**

| **Variable** | **Univariate analysis** | | |  | **Multivariate analysis** | | |
| --- | --- | --- | --- | --- | --- | --- | --- |
|  | **HR** | **95% CI^§^** | ***P*** |  | **HR** | **95% CI** | ***P*** |
| **Progression-free survival (PFS)** | | | | | | | |
| Age  < 65 versus ≥ 65 years | 0.89 | 0.566-1.401 | 0.5903 |  |  |  |  |
| Gender  male versus female | 0.84 | 0.500-1.429 | 0.4812 |  |  |  |  |
| DDR alteration  yes versus no | 0.60 | 0.363-0.979 | **0.0500** |  |  |  |  |
| Core DDR alteration  yes versus no | 0.28 | 0.080-0.983 | 0.1673 |  |  |  |  |
| Actionable alteration  yes versus no | 0.63 | 0.387-1.024 | 0.0675 |  |  |  |  |
| Targeted treatment  yes versus no | 0.46 | 0.163-1.321 | 0.0680 |  |  |  |  |
| **Overall survival (OS)** | | | | | | | |
| Age  < 65 versus ≥ 65 years | 0.85 | 0.514-1.396 | 0.4926 |  |  |  |  |
| Gender  male versus female | 0.90 | 0.485-1.660 | 0.7113 |  |  |  |  |
| DDR alteration  yes versus no | 0.78 | 0.440-1.376 | 0.4019 |  |  |  |  |
| Core DDR alteration  yes versus no | 0.40 | 0.094-1.665 | 0.3393 |  |  |  |  |
| Actionable alteration  yes versus no | 0.80 | 0.464-1.393 | 0.4419 |  |  |  |  |
| Targeted treatment  yes versus no | 0.32 | 0.075-1.396 | **0.0240** |  | 0.07 | 0.011-0.435 | **0.004** |
|  |  |  |  |  |  |  |  |

^§^ CI, confidence interval
